# A Web-Based Decision Support System for Assessing Regional Water-Quality Conditions and Management Actions^1^

**DOI:** 10.1111/j.1752-1688.2011.00573.x

**Published:** 2011-10

**Authors:** Nathaniel L Booth, Eric J Everman, I-Lin Kuo, Lori Sprague, Lorraine Murphy

**Keywords:** assessment, communication, environmental management, water-quality planning, Geographical Information System, geospatial analysis, Internet, decision support system, simulation, water

## Abstract

**Abstract:**

The U.S. Geological Survey National Water Quality Assessment Program has completed a number of water-quality prediction models for nitrogen and phosphorus for the conterminous United States as well as for regional areas of the nation. In addition to estimating water-quality conditions at unmonitored streams, the calibrated SPAtially Referenced Regressions On Watershed attributes (SPARROW) models can be used to produce estimates of yield, flow-weighted concentration, or load of constituents in water under various land-use condition, change, or resource management scenarios. A web-based decision support infrastructure has been developed to provide access to SPARROW simulation results on stream water-quality conditions and to offer sophisticated scenario testing capabilities for research and water-quality planning via a graphical user interface with familiar controls. The SPARROW decision support system (DSS) is delivered through a web browser over an Internet connection, making it widely accessible to the public in a format that allows users to easily display water-quality conditions and to describe, test, and share modeled scenarios of future conditions. SPARROW models currently supported by the DSS are based on the modified digital versions of the 1:500,000-scale River Reach File (RF1) and 1:100,000-scale National Hydrography Dataset (medium-resolution, NHDPlus) stream networks.

## Introduction

Water-resource managers and policy makers have long valued federal, state, and local stream monitoring data (USEPA, 2010) as a basis for defining the status and trends of the quality of the nation's water resources. While these monitoring data are highly informative and generally widely available from public databases on the Worldwide Web (U.S. Environmental Protection Agency, STORET. http://www.epa.gov/storet/index.html, *accessed* June 2010; U.S. Geological Survey, National Water Information System: Web Interface. http://waterdata.usgs.gov/nwis, *accessed* September 2009), the data do not provide a complete spatial coverage of United States (U.S.) water bodies to support water quality decision making. To compensate for these deficiencies, water managers and scientists have used the predictive capabilities of hydrological and chemical models. These models typically use mathematical process representations in addition to monitoring and geospatial data to estimate water-quality conditions over time and space (Alexander *et al.*, 2008). By combining current water-quality data with predictive models, “what-if” scenario testing and policy impact analysis can also better inform management and policy decisions than data alone (Graffy and Booth, 2008). However, it is difficult for decision makers to readily access model information and use models directly to evaluate alternative scenarios. Modeling experts are commonly required to assist with requests from managers, either to communicate the nuances of the model and software or to provide the intensive data input requirements for running model simulations. This makes the use of predictive models cumbersome and costly for decision makers and reinforces the sense that models are far from a mainstream tool to assist deliberation. SPAtially Referenced Regressions On Watershed Attributes (SPARROW) is a source-transport model that provides the capability to predict constituent loads, concentration, and yield in streams over regional and continental spatial scales. The model has been previously used to simulate nutrient conditions in the Mississippi River basin (Alexander *et al.*, 2008), the Chesapeake Bay watershed (Preston and Brakebill, 1999), and other major drainages of the U.S. (e.g., Moore *et al.*, 2004; Anning *et al.*, 2007; Hoos and McMahon, 2009). The U.S. Geological Survey (USGS) National Water Quality Assessment (NAWQA) Program has also recently developed SPARROW models for six large regions of the conterminous U.S. (referred to as “Major River Basins,” or MRBs) (see Preston *et al.*, this issue, for a summary of the modeling studies). The MRB models can provide baseline information to inform water-resource investigations and management for most watersheds in the U.S., including a model platform that can support the evaluation of land and water management scenarios (e.g., “what are the downstream effects on nutrient loads of managed nutrient reductions in watershed X?”). However, the use of these or other SPARROW models by managers to obtain baseline model output or to support detailed simulations for scenario evaluation typically entails substantial training and expertise.

In this article, we describe a new approach for making SPARROW model results and applications readily accessible to a broad audience through a web-based decision support system (DSS). Others have found that the structure of SPARROW lends itself well to DSSs and have developed application programming interfaces to be used with a calibrated SPARROW model for running predictions (Goodall *et al.*, 2010). The power of this new DSS is its ability to provide model-based decision support that is easy to use and comprehend using map-based displayed results. The DSS also allows managers and scientists to collaborate on the creation of modeled scenarios by running predictions on pre-calibrated models with adjusted model parameters (like land use or fertilizer, e.g.). The DSS and underlying software framework that we present is intended to make running sophisticated SPARROW model simulations easier by combining familiar website user interface controls with a powerful computer server infrastructure. This paradigm places new capabilities in the hands of decision makers and water-quality planners and managers in ways that previously were not available, but it does not remove the importance of working with experienced SPARROW modelers who understand the nuances, strengths, and weaknesses of the models. The DSS also illustrates innovations in the information technology field that allow for a flexible and robust web-based decision support framework that applies beyond the SPARROW model and could be useful for other modeling systems. The DSS removes desktop software dependencies, simplifies scenario testing, and provides a map interface. All of these features are validated to the original nominal model behind the scenes, yet delivered through a simplified user interface in a web-browser over an Internet connection.

The SPARROW DSS can be accessed at the following Internet address: http://water.usgs.gov/nawqa/sparrow/dss/.

## Design of the SPARROW DSS

The SPARROW model establishes a relation between instream constituent estimations and the environmental characteristics of the contributing land area at a group of monitoring stations to estimate water-quality conditions throughout a network of stream reaches (Schwarz *et al.*, 2006). Model estimates of constituent load, yield, or concentration in all stream reaches, including those that have not been monitored previously, can help identify specific locations where water-quality problems may be present.

The SPARROW DSS runs calibrated SPARROW models within a web browser without requiring users of the system to install special software or to take special training. The target users for the DSS are water-resources managers and researchers with a general familiarity of hydrologic principles. The power of the DSS is its ability to provide model-based decision support that is easy to use, is easy to comprehend using the map-based displayed results, and provides the ability to collaborate on the creation of modeled scenarios.

SPARROW Attributes models that conform to supported stream reach networks (Brakebill *et al.*, this issue) can be uploaded to a common database repository that supports the SPARROW DSS. Once loaded into the repository, models are available through the DSS. Models currently available in the DSS are described in Table 1.

**TABLE 1:** Initial Models Available From SPARROW Model Archive

Model Name	Geographic Focus	Base Year	River Network	Citation
National Total Nitrogen	Coterminous United States	1992	Enhanced River Reach File 2.0 (E2RF1) (1:500K) (Nolan *et al.*, 2002)	Alexander *et al.* (2008)
National Total Phosphorus	Coterminous United States	1992	Enhanced River Reach File 2.0 (E2RF1) (1:500K) (Nolan *et al.*, 2002)	Alexander *et al.* (2008)
MRB1 Total Nitrogen	New England and Mid-Atlantic Regions	2002	NHDPlus (1:100K) (Horizon Systems Corporation, 2009)	Moore *et al.* (this issue)
MRB2 Total Nitrogen	South Atlantic-Gulf and Tennessee Regions	2002	Enhanced River Reach File 2.0 (E2RF1) (1:500K) (Nolan *et al.*, 2002)	Hoos and McMahon (2009)
MRB2 Total Phosphorus	South Atlantic-Gulf and Tennessee Regions	2002	Enhanced River Reach File 2.0 (E2RF1) (1:500K)(Brakebill *et al*., this issue)	Garcia *et al.* (this issue)
MRB3 Total Nitrogen	Laurentian Great Lakes Regions	2002	Enhanced River Reach File 2.0 (E2RF1) (1:500K) (Brakebill *et al*., this issue)	Robertson and Saad (this issue)
MRB3 Total Phosphorus	Laurentian Great Lakes Regions	2002	Enhanced River Reach File 2.0 (E2RF1) (1:500K) (Brakebill *et al*., this issue)	Robertson and Saad (this issue)
MRB4 Total Nitrogen	Missouri River Basin	2002	Enhanced River Reach File 2.0 (E2RF1) (1:500K) (Brakebill *et al*., this issue)	Brown *et al.* (this issue)
MRB4 Total Phosphorus	Missouri River Basin	2002	Enhanced River Reach File 2.0 (E2RF1) (1:500K) (Brakebill *et al*., this issue)	Brown *et al.* (this issue)
MRB5 Total Nitrogen	Lower Mississippi River and Texas-Gulf Basins	2002	Enhanced River Reach File 2.0 (E2RF1) (1:500K) (Brakebill *et al*., this issue)	Rebich *et al.* (this issue)
MRB5 Total Phosphorus	Lower Mississippi River and Texas-Gulf Basins	2002	Enhanced River Reach File 2.0 (E2RF1) (1:500K) (Brakebill *et al*., this issue)	Rebich *et al.* (this issue)
MRB7 Total Nitrogen	Pacific Northwest Region	2002	Enhanced River Reach File 2.0 (E2RF1) (1:500K) (Brakebill *et al*., this issue)	Wise and Johnson (this issue)
MRB7 Total Phosphorus	Pacific Northwest Region	2002	Enhanced River Reach File 2.0 (E2RF1) (1:500K) (Brakebill *et al*., this issue)	Wise and Johnson (this issue)

Note: SPARROW, SPAtially Referenced Regressions On Watershed Attributes; MRB, Major River Basin (see Preston *et al.*, this issue).

A calibrated SPARROW model can be used for a variety of information needs, and the DSS is designed to facilitate those uses. The model estimates constituent load, yield, and concentration in all stream reaches within the modeled stream reach network and provides error estimates for each. It also identifies important sources contributing to the constituent load in a stream reach and traces the transport of these constituents downstream through the reach network to receiving water bodies such as reservoirs and estuaries. Inputs from different constituent sources can be altered to investigate potential changes in the quality of water in individual stream reaches and in downstream receiving water bodies under hypothetical conditions.

The general design of the SPARROW DSS user interface and computer application is based on the predictive and scenario-based capabilities of the SPARROW model. The user interface is designed with a map display that is controlled and investigated according to specific SPARROW model functions through a familiar web mapping layout. The computing architecture is an integration of separately functioning modular components that provide a scalable and extensible platform for accommodating additional models and decision support capabilities as they are developed.

## User Interface Design

The SPARROW DSS is organized around six primary functions (Table 2). Those functions are implemented in the user interface as follows. First, model estimates can be described spatially by mapping model-estimated metrics such as the mean annual amount of total load of a constituent and derivative metrics such as mean annual constituent concentration for each river reach. Second, model uncertainty provides an estimate of the prediction error associated with the mean annual amount of total load of the constituent based on the model calibration. The third and fourth functions describe model inputs and stream network characteristics geospatially through the use of digital mapping. The DSS provides the capability of describing the spatial pattern of important variables such as constituent sources that drive the water-quality conditions. (See Wieczorek, 2009, for a description of many source variables used in SPARROW models.) The fifth function of the DSS, downstream effects, allows the user to evaluate the delivery of constituents to downstream water bodies by traversing the river network and considering modeled load attenuation processes. The first five primary functions of the DSS can be extended to assist users in testing how changing landscape or source inputs can influence water-quality conditions locally and downstream and how simulated source reductions or increases might impact the goals of potential management actions (Simulations). Users can test these management scenarios by developing a source management plan and mapping or graphing the predicted downstream response in loads. Simulations are limited to changing values of model parameters represented within each model. Other variables cannot be introduced into an existing calibrated SPARROW model for the purposes of running scenarios through the DSS.

**TABLE 2:** Six Functions of the SPARROW DSS and Related Data Series

	Units	Description
Model estimates		
Total load	kg/year	The mean annual load of the constituent leaving each stream reach, as predicted by the model. The load reflects the accumulated mass of the constituent contributed by all or individual sources in the total drainage area upstream of the reach outlet. The load includes the effects of instream attentuation processes in all upstream reaches. The mean annual load is a standardized measure of the constituent mass in the stream that reflects the mean quantities of mass that are likely to occur during a specified base year under long-term mean streamflow conditions
Incremental load	kg/year	The mean annual load of the constituent entering the stream reach from sources in the incremental drainage area of the reach. The load value reflects the effects of instream attentuation processes associated with one half of the reach time of travel (this assumes that the load is at approximately the center of reach on average)
Concentration	mg/l	The average concentration of the constituent in the reach (in units of volume per time; mg/l). This is calculated by dividing the total load by the mean annual flow of the reach
Incremental yield	kg/km^2^/year	The incremental load divided by the incremental drainage area of the reach
Model uncertainty		
Standard error of total load	kg/year	An estimate of the prediction error associated with the total load, based on the model calibration is provided
Standard error of incremental load	kg/year	An estimate of the prediction error associated with the total load, based on the model calibration is provided
Model inputs		
Nutrient sources	Various	The amount of a particular source added in the reach's drainage area, for example fertilizer applied in an agricultural area. Sources may also be represented by land units such as urban land. For these data series, you must choose an individual source to map
Stream network characteristics		
Incremental area	km^2^	The “incremental” area is the area that drains directly to the reach without passing through another reach. This area is independent of the drainage area associated with upstream reaches
Streamflow	cubic feet per second	The mean annual streamflow of the reach
Downstream effects		
Delivery fraction	Percent	The fraction of the load leaving a reach that arrives at the downstream end of a selected target reach without any removal by natural attenuation processes (e.g., long-term storage; denitrification). You must choose a target reach if you select this series
Incremental delivered load	kg/year	The incremental load associated with a stream reach that arrives at the downstream end of a selected target reach. You must choose a target reach if you select this series
Total delivered load	kg/year	The total load associated with a stream reach that arrives at the downstream end of a selected target reach
Incremental delivered yield	kg/km^2^/year	The incremental yield associated with a stream reach that arrives at the downstream end of a selected target reach
Simulations		
Absolute change from original model	Absolute value	The absolute value of the change from the original predicted model estimates or downstream effects compared to the predicted model estimates or downstream effects with altered model inputs. For this comparison data series, you must simulate a management scenario with constituent source reductions or increases
Percent change from original model	Percent	The percent change from the original predicted model estimates or downstream effects compared to the predicted model estimates or downstream effects with altered model inputs. For this comparison data series, you must simulate a management scenarios with constituent source reductions or increases

Notes: SPARROW, SPAtially Referenced Regressions On Watershed Attributes; DSS, decision support system. The DSS displays streamflow in the English units of cubic feet per second rather than SI units.

The SPARROW model and the SPARROW DSS break down constituent load estimates into incremental or total catchment areas. Incremental catchments are the small, local watersheds draining directly to a given stream reach; total catchments are the incremental catchment plus all of the upstream catchments draining to a stream reach. The constituent load estimates from either the incremental or total catchment area can be mapped in the DSS for all stream reaches in the reach network. Information discretized by incremental catchment preserves detail on the spatial distribution of source and transport attributes, whereas information accumulated over the total catchment enables comparison of conditions among larger basins.

**FIGURE 1:**
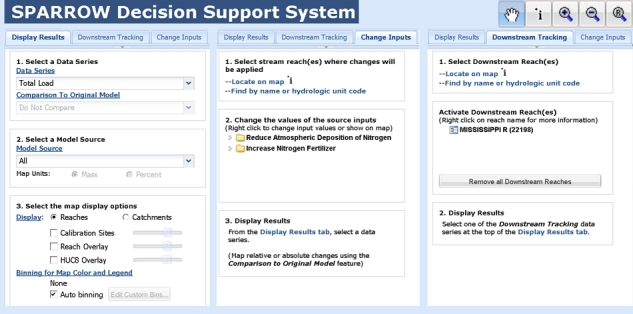
The Arrangement of Prediction Display Options and Main Toolbar of the Decision Support System (DSS).

The SPARROW DSS user interface is designed to support the six primary functions of the tool. The interface accepts user input through a series of input menus, and a map pane reflects SPARROW model output based on user choices. Three tabs, “Display Results,”“Downstream Tracking,” and “Change Inputs” and a main toolbar assemble the user input sections (Figure 1). “Display Results” manages the SPARROW model data series displayed on the map, including total load, mean annual concentration, and magnitude of model source terms. “Change Inputs” manages the simulation function of the DSS to define and test “what if” scenarios by organizing groups of reaches with altered source inputs. Users create scenarios by selecting stream reaches or hydrologic units (Steeves and Nebert, 1994) and adjusting the source input values manually; scenarios can be saved on a personal computer and uploaded to the tool for future analysis. The DSS has the capability to map all the available data series for a “what if” scenario, with the additional option to compare the difference between simulation estimates and the original model estimates by absolute value or percent change. “Downstream Tracking” is used for managing local river reaches of interest. Target reaches included in this section of the DSS are often designated as river mouths to summarize estuary or lake inputs, but any river reach within the stream network may be selected as a target reach to view how it is affected by upstream influences. Standard web mapping controls in the map toolbar of the DSS such as Identify Reach and zoom capabilities allow for further exploration of the SPARROW model source inputs and prediction results displayed on the map in combination with additional stream network attributes.

All functions of the DSS are oriented around the spatial display of model estimations on a regional network of river reaches. The map of the modeled region, which is designed to display individual reaches or catchments, makes up the majority of the application interface with an explanation containing the type of data series being mapped (Table 2) and respective units, map categories and scale. The map is continuously updated to reflect any changes the user makes to the prediction display options. An example of a typical use case with reference to the general layout of the application interface and map display is described below.

The SPARROW DSS offers other features for analysis. A variety of background map layers – including political boundaries, elevation, land cover, and aerial imagery – can be displayed. The location of calibration sites for each model can be overlaid on any mapped data series. Documentation for the use and theory of the SPARROW model and for the calibration of the individual regional models is available on each model page. Finally, the original and adjusted model output can be exported in comma-separated, tab-delimited, or Microsoft Excel format for viewing or use in external applications.

## Computing Architecture Design

**FIGURE 2:**
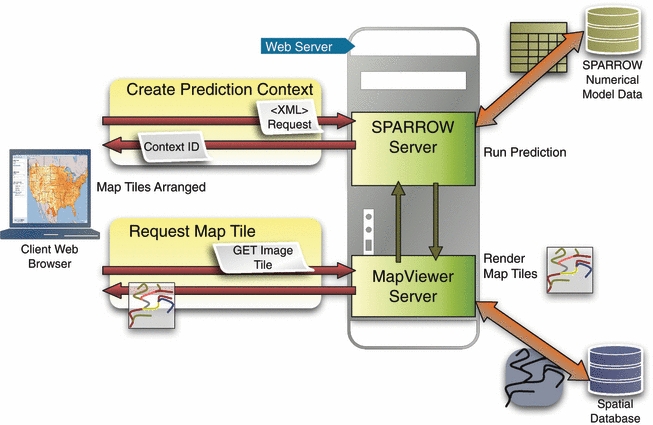
Decision Support System (DSS) Model Simulation Workflow.

The DSS is built on a “service-oriented computing architecture,” which allows for efficient software development and operation. Service-oriented architecture is a design philosophy that separates software into modular components such that each component can be developed and maintained separately, while the functions of each component can be integrated to produce a cohesive and robust product (Kaye, 2003). The DSS framework takes user input, runs a SPARROW model prediction, and produces water-quality maps, statistical summaries, and graphics via two modular database-backed web services – the SPARROW prediction service that runs the SPARROW model and the MapViewer mapping service that generates map images of stream reaches and catchments (Figure 2). Additionally, water-quality maps are overlaid on background maps via web services from the USGS National Map and other Internet resources (USGS, 2010). This type of framework design provides a strong basis for implementation of a computationally scalable web-based spatial DSS (Ostlander, 2004). The SPARROW DSS is made up of two servers, two databases, and a client that runs in the user's web browser. The first server is called the prediction server and is responsible for generating SPARROW predictions based on a set of parameters specified to generate predictions from a SPARROW model. The set of parameters is called the prediction context and is defined using an Extensible Markup Language (XML) document (Bray *et al.*, 2008). The prediction server loads SPARROW model data from an Oracle database, which stores all the calibrated SPARROW models in a common and unified repository.

When a model simulation is requested, the prediction server calculates an estimated load for each individual stream reach and accumulates the load down the river network by combining upstream loads. Given that the loads of individual stream reaches are accumulated with progression through the stream network to define downstream loads, the individual stream loads are referred to as “incremental” (Schwarz *et al.*, 2006). Load is estimated at the end of each reach segment after incorporating an instream decay function. This overall load accumulation process is computationally intensive, executing more than 1.2 million calculations per national simulation of a model built from modified versions of River Reach File (Brakebill *et al.*, this issue).

The second server is called the mapping server. The mapping server is responsible for drawing the map images displayed in the client web browser and is based on Oracle's 10G MapViewer product (Oracle, 2009) with an extension that provides predicted values from the prediction server. To render the map image, the map server processes two data components: the predicted values from the prediction server and the geospatial data (points, lines, and curves) that define the geometry of the rivers. The mapping server requests the predicted values from the prediction server and loads the geospatial data from an Oracle Spatial database. To render a map image, MapViewer merges these two datasets, appropriately coloring each river reach based on the styling rules defined in the user's web browser.

The portion of the DSS that runs in the web browser uses standard web technologies and components to allow the user to choose options, interact with the map, and communicate the user's requests back to the web servers. Standard website components provide a familiar user interface, which are being used increasingly in common web-based productivity tools such as web email and web mapping applications.

The prediction server and the mapping server together make up the DSS server. Programs are written in Java and run within a Java 2 Enterprise Edition (J2EE) host environment (Sun Microsystems, 2009). Oracle MapViewer and database are both commercial products, while the J2EE host environment could be any one of a number of free or commercial options meeting the J2EE specification (Sun Microsystems, 2009).

The central workflow of the DSS application is the model simulation mapping workflow, which takes place after the user has updated a scenario or changed mapping options, and has requested that the map be updated. In this workflow, shown in Figure 2, the client browser registers a prediction context with the server, then requests map images from the server based on that prediction context. When the map images have been requested from the MapViewer Server, the model calculations are executed on the SPARROW Server based on model information (metadata), including model calibration coefficients, stored in the SPARROW Model Database. When the model calculations are complete, the results are sent back to the MapViewer Server where stream reaches stored in the Spatial Database are displayed.

While the primary objective of the DSS is to display SPARROW simulation results on a map, SPARROW predictions can be generated without a map display. Because the Prediction Server and Mapping Servers are distinct, prediction contexts can be submitted directly to the Prediction Server, bypassing the mapping component to deliver model exports in numerical form for use in other applications or models, such as a lake or estuary water-quality model. Numerical exports reference stream reaches by reach name or unique reach identifiers to facilitate model linkage.

## Examples of SPARROW DSS Applications

**FIGURE 3:**
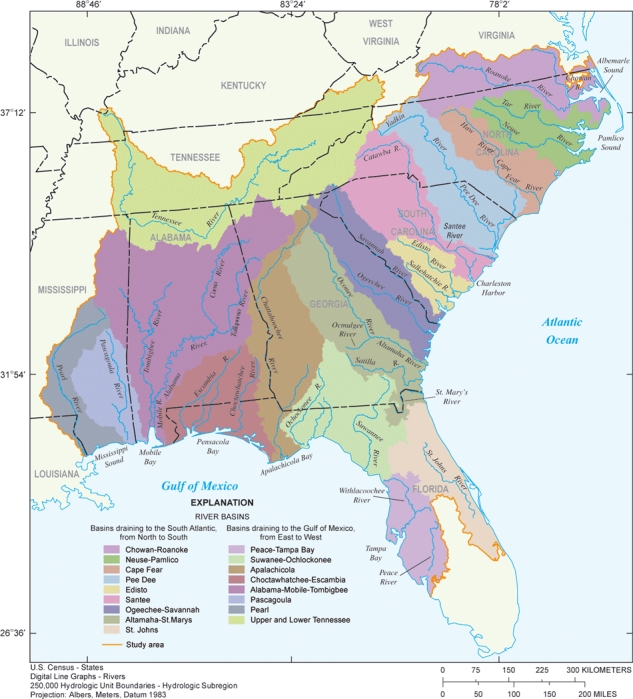
The MRB2 Study Area, Which Includes Major River Basins Draining to the South Atlantic Coast, Eastern and Central Gulf Coast, and the Tennessee River. Modified From Garcia et al. (this issue).

Examples and uses of the DSS are presented for the South Atlantic-Gulf and Tennessee River basins (SAGT) based on a calibrated total nitrogen SPARROW model described in Hoos and McMahon (2009). The SAGT region includes river basins draining to the South Atlantic coast, Eastern and Central Gulf coast, and the Tennessee River (Figure 3).

## Description of Water-Quality Conditions

**FIGURE 4:**
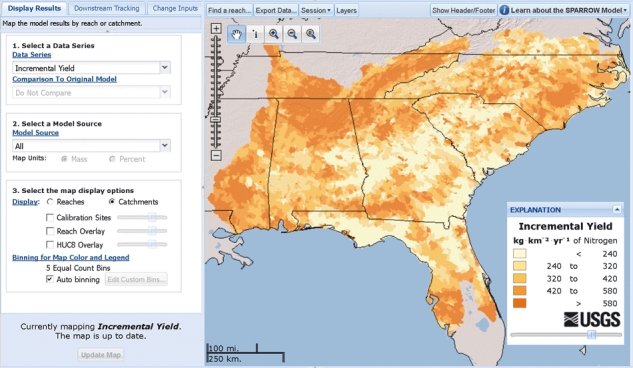
Incremental Yield of Total Nitrogen in the South Atlantic-Gulf and Tennessee River Basins (SAGT) Drainage Area, in kg/km^2^/year.

The model-estimated incremental yield (in kg/km^2^/year) of total nitrogen in streams of the SAGT region are displayed in Figure 4. The mapping capabilities of the DSS highlight the spatial patterns of high and low incremental yield of total nitrogen. Stream reaches with relatively high incremental nitrogen yield are distributed throughout the region, but are more concentrated in Mississippi, central and eastern Tennessee, northern Alabama, eastern North Carolina, and west central Florida. Stream reaches with relatively low nitrogen yield also are present throughout the region, but are more concentrated in central and southeastern Georgia, and the Florida panhandle (Hoos and McMahon, 2009).

## Allocation of Nutrient Sources

The spatially distributed structure of the SPARROW model enables separate quantification of individual constituent source contributions and constituent loss in streams and reservoirs during downstream transport (instream and reservoir decay). This model capability allows comparison between the mass from individual constituent sources input within an incremental catchment and the load from that source in the catchment's corresponding stream reach. It also allows comparison between the incremental load from an individual source and the overall incremental load from all sources. Moreover, quantification of land-to-water delivery and instream and reservoir decay allows the instream load for any given stream reach to be traced back to individual constituent sources in each of the upstream catchments contributing load to that reach. As a result, the contribution of each individual constituent source to the total instream load (or yield) also can be quantified. Identification of the largest constituent sources contributing to instream load is often a key component of cost-effective management of water resources.

**FIGURE 5:**
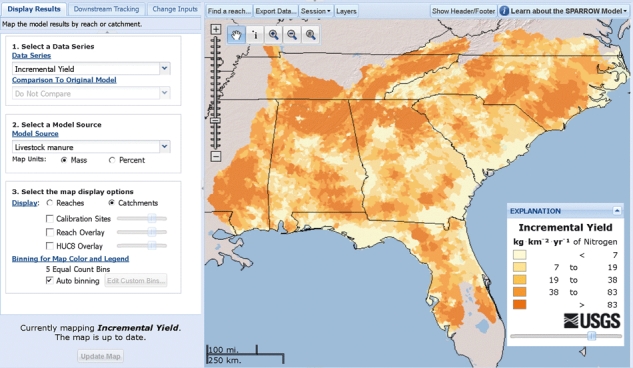
Incremental Yield of Total Nitrogen From Livestock Manure in the South Atlantic-Gulf and Tennessee River Basins (SAGT) Region, in kg/km^2^/year.

The DSS can map the estimated load of an individual source as if all other sources are turned off, either as the incremental load or total load. In the SAGT model, livestock manure is one of the significant sources of nitrogen. The contribution of this one source to stream nitrogen loads can be selected in the DSS through a series of controls. A map displays the proportion of incremental yield from livestock manure for all reaches, indicating regions and catchments with high contributions from that source to the river network (Figure 5). Areas with relatively high incremental yield from livestock manure are in parts of central Mississippi, northern Alabama, central and eastern Tennessee, northern Georgia, and eastern North Carolina. A comparison of the incremental yield from all sources (Figure 4) to the incremental yield from livestock manure (Figure 5) illustrates that the contribution of livestock manure to the overall incremental yield in some of these areas is high.

**FIGURE 6:**
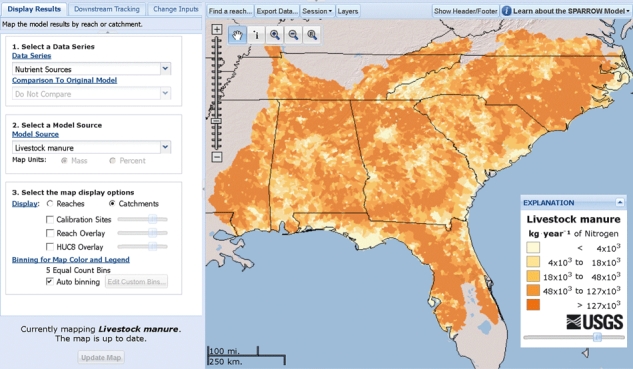
The Amount of Nitrogen in Livestock Manure Input Within Each Incremental Catchment in the South Atlantic-Gulf and Tennessee River Basins (SAGT) Region, in kg/km^2^/year.

The values in Figure 5 also can be compared to the amount of nitrogen derived from livestock manure input on the land surface within each incremental catchment (Figure 6) – catchments with large amounts of manure input correspond to the stream reaches with the highest incremental yield from manure (Figure 5). However, a comparison of the respective values in Figures 5 and 6 indicates that only a small portion of the nitrogen in manure input in a catchment reaches the streams in the SAGT region.

**FIGURE 7:**
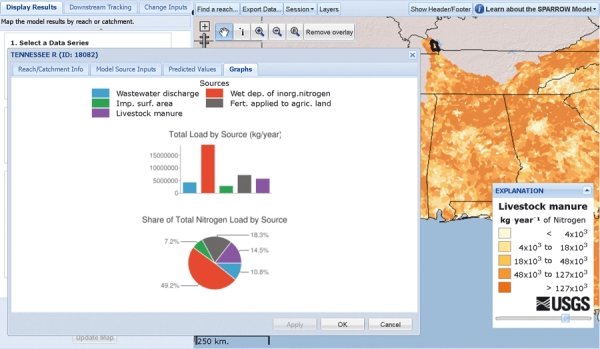
The Contributions of Individual Sources to the Total Load of Total Nitrogen in Stream Reach 18,082 (from Brakebill *et al.*, this issue) of the Tennessee River (top bar plot in kg/year, bottom pie chart in percent of total load).

The DSS can provide more detailed information on the relative contribution of individual sources to the total instream load for a particular stream reach. In Figure 7, the total instream load of total nitrogen in reach number 18,082 in the western part of the SAGT region is displayed along with information on the relative amount of nitrogen originating from all sources used in the model. In this reach, source 2 (wet deposition of inorganic nitrogen) is the largest contributor to the total instream load of nitrogen. Other information about the reach, such as hydrologic characteristics (from Brakebill *et al.*, this issue), model inputs, and model estimates, is available using various options in the DSS.

## Evaluation of Nutrient Delivery to Downstream Water Bodies

As the amount of instream or reservoir decay that occurs in each stream reach is quantified in the SPARROW model, the fraction of the load in each reach that is ultimately delivered to a downstream target water body can be determined and displayed in the DSS. The delivery fraction, together with the magnitude of the load from individual sources or all sources combined, can provide insight into the geographic areas and individual sources contributing the most to the constituent load entering a target water body such as an estuary, reservoir, or a stream confluence.

**FIGURE 8:**
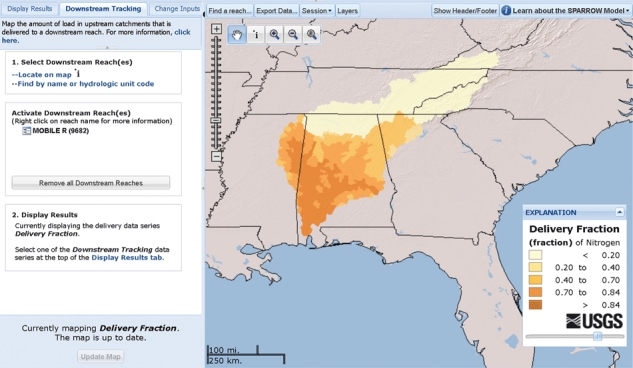
The Fraction of Total Nitrogen Load That Arrives to Mobile Bay From Each Stream Reach in Its Drainage Area.

In Figure 8, the last stream reach on the Mobile River before it enters Mobile Bay (reach 9,682) has been set as the target reach to evaluate delivery to Mobile Bay from its drainage area. Mobile Bay itself cannot be set as the target reach because estuaries are not included in the reach network utilized by the SPARROW model. Stream reaches having a large percentage of their incremental loads being delivered directly to Mobile Bay (defined in the DSS as delivery fraction) include those in close proximity to the bay, where the short travel times leave relatively little opportunity for instream or reservoir loss. Stream reaches with a low percentage of their loads delivered to the bay include those streams far from the bay and those upstream of reservoirs that have the potential to enhance reservoir-based attenuation processes. Streams in the Tombigbee River basin in northeastern Mississippi and western Alabama generally have higher delivery fractions than many of the streams in the Alabama River basin in central and eastern Alabama and northwestern Georgia, in part due to reservoir decay behind the series of locks and dams on the Alabama, Coosa, and Tallapoosa Rivers (Figure 8; see Figure 3 for the location of tributaries to Mobile Bay).

**FIGURE 9:**
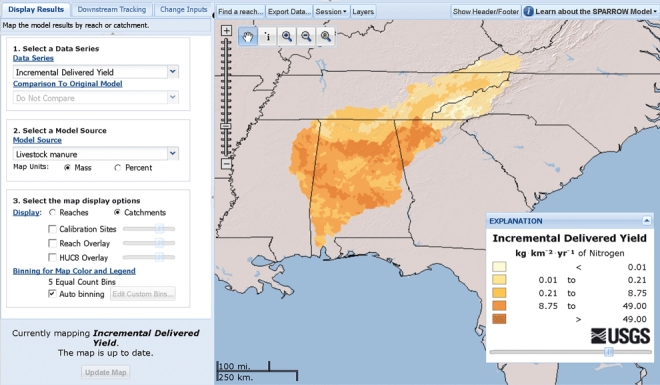
Incremental Delivered Yield of Total Nitrogen to Mobile Bay From Each Stream Reach in Its Drainage Area, in kg/km^2^/year.

The incremental delivered yield of total nitrogen, displayed in Figure 9, is dependent on the magnitude of the incremental yield (Figure 4) as well as the delivery fraction (Figure 8) that defines the amount transported to the targeted downstream reach. Areas delivering the highest nitrogen yield to Mobile Bay include the upper Tombigbee and central Alabama River basins in northeastern Mississippi and central Alabama (Figure 9), where incremental nitrogen yield and delivery fractions are high. Areas delivering the lowest yield to Mobile Bay include the tributaries to the lower Tombigbee River basin in southwestern Alabama, where delivery fractions are high but incremental yields are relatively low, and the Coosa and Tallapoosa River basins, where incremental yields are relatively high but delivery fractions are low (Figure 9).

## Simulation of Scenarios of Altered Nutrient Inputs

A calibrated SPARROW model also can be used to estimate constituent load, yield, or concentration under a set of altered inputs or conditions. The ability to portray possible future conditions for specified inputs is one of the most powerful uses of and reasons for constructing models, as there are commonly no alternative methods for conducting controlled experiments on complex systems (Schwarz *et al.*, 2006). For example, the alteration of nutrient inputs could be part of an evaluation of future nutrient-reduction strategies or land-use changes, providing an estimate of the water-quality benefits that might be realized from a given management action.

**FIGURE 10:**
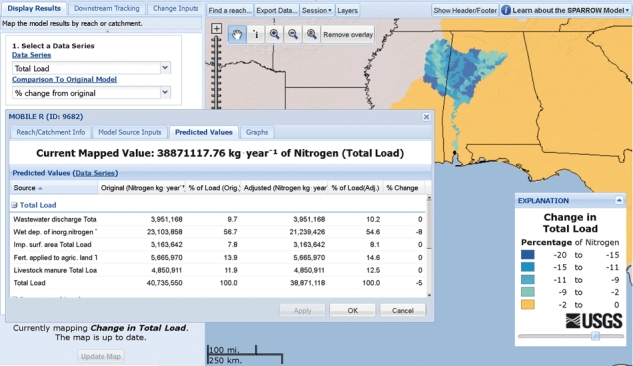
Percent Change in Total Load of Total Nitrogen in the Lower Mobile River Basin After a Hypothetical 20% Reduction in Wet Deposition of Inorganic Nitrogen in the Black Warrior-Tombigbee HUC6, Relative to the Original Model Estimates. The original and adjusted (“treated”) loads entering Mobile Bay from individual and combined sources is shown in the inset table.

Conditions can be adjusted in the DSS for an individual reach or a group of reaches for either one or multiple source inputs. In Figure 10, wet deposition of inorganic nitrogen was decreased by 20% in the Black Warrior-Tombigbee HUC6 (6-digit hydrologic unit code) drainage area within the northwestern Mobile Bay basin, where incremental loads delivered to Mobile Bay were relatively high in the original model (Figure 9). It is clear from the map that nitrogen decreased from <14 to as much as 20% in individual stream reaches in the Black Warrior-Tombigbee HUC6 drainage area, with more substantial percentage decreases in reaches for which the source share of atmospheric deposition is largest (Figure 10). The total load of nitrogen entering Mobile Bay from the Mobile River was estimated to decrease approximately 5% in this scenario (Figure 10).

**FIGURE 11:**
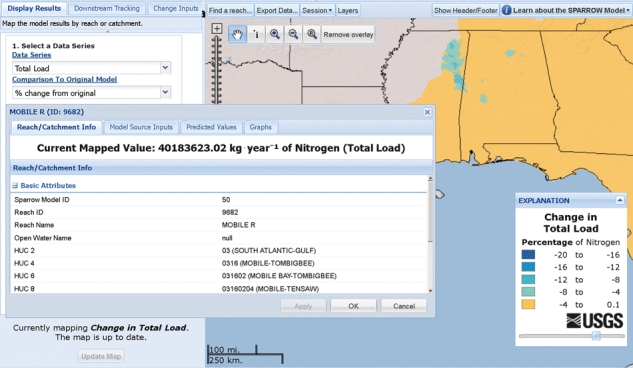
Percent Change in Total Load of Total Nitrogen in the Lower Mobile River Basin After a Hypothetical 20% Reduction in Fertilizer Applied to Agricultural Land in the Black Warrior-Tombigbee HUC6, Relative to the Original Model Estimates. The original and adjusted (“treated”) loads entering Mobile Bay from individual and combined sources is shown in the inset table.

In Figure 11, fertilizer applied to agricultural land was decreased by 20% in the Black Warrior-Tombigbee HUC6. The total load of nitrogen decreased from <1 to as much as 10.4% in individual stream reaches in this HUC6, with more substantial percentage decreases in reaches for which the source share of fertilizer is largest (Figure 11). The total load of nitrogen entering Mobile Bay from the Mobile River was estimated to decrease only about 1% in this scenario (Figure 11), a smaller reduction than a 20% reduction in atmospheric deposition of inorganic nitrogen (5%). The difference between the two scenarios suggests that a greater percentage reduction in fertilizer applied to agricultural land than in wet deposition of inorganic nitrogen would likely be needed to achieve the same reduction in the total load entering Mobile Bay. Users of the DSS may also want to consider other likely contemporaneous changes in sources within a single counterfactual scenario – for example, a decrease in fertilizer applied to agricultural land may be associated with an increase in impervious surface area as a consequence of urban development, and both could be considered together.

## Sharing and Publishing Model Scenarios

The DSS enables users to build and share complex scenarios across their community through *predefined scenarios*. The ability to easily save, share, and publish scenarios allows the DSS tool to facilitate collaboration and makes the application more approachable to casual users. Predefined scenarios encapsulate two distinct concepts, the simulation instructions needed to generate predictions from the model and the information required to describe the state of the application. The model simulation instructions, called the prediction context, contain the adjustments to model sources, the target reach selections and specifies the data series to generate. The predefined session contains the prediction context *and* all of the relevant application states, which includes the map categories, the map scale and viewing area, and the background map layers and overlays.

Predefined scenarios can be recalled through several means. Authorized users (primarily model authors) can publish predefined scenarios to the DSS using an administrative web-based form. After approval, a URL containing the unique identifier for that scenario is then created that will link directly to the application with that predefined scenario loaded. This provides a convenient mechanism for linking into the DSS from an online publication describing findings of the model or from other web resources describing the model. Once opened in the DSS, the scenario can be further investigated or manipulated using the tools described in the User Interface Design section. The scenario described in the section above can be loaded by accessing the following URL: http://cida.usgs.gov/sparrow/map.jsp?model=50&session=example.

While storing predefined scenarios on the DSS server is only available to authorized users, any user of the DSS can save and share model scenarios through predefined session files. Once the user has a scenario defined and has chosen mapping options that highlight their finding, a predefined session can be downloaded from the DSS as a text file. The predefined session file can then be distributed among colleagues or a broader community via email or through a website or blog, allowing students, researchers, and others to collaborate and share results.

For a user working with the DSS for the first time, following a link to a predefined scenario provides an easy way to “learn by doing,” demonstrating advanced application features without requiring the user to learn them first. Educators or instructors who want to use SPARROW models in their curriculum can also distribute their own predefined session file for their students to work from.

Future versions of the DSS may include the ability to more easily manipulate predefined session files outside of the DSS. For complex scenarios that include custom adjustments to input sources at hundreds or thousands of stream reaches, constructing a predefined session file outside of the DSS may prove more convenient.

## Conclusions

Hydrologic model computer simulations have provided insight to policy makers and resource managers for decision making for decades, but they have typically not been provided in a form that managers could use without the support of technical experts. The DSS approach presented here addresses this problem, which should make predictive water-quality models more available as a standard decision-making tool. With many modeling software packages, running these simulations requires firsthand knowledge of the calibration process, the nuances of the modeling software package, and all related data processing. Additionally, proprietary software and increasingly more powerful computer servers are often needed to run simulations. Because of these limitations, many models are beyond the reach of many users, and simulations beyond a modeling project's initial scope are frequently not pursued. Thus, resources expended to collate and process observational data and to calibrate the model are not leveraged to the extent possible. A web-based DSS can assuage many of these issues that prevent subsequent model simulations.

The SPARROW DSS provides a tool for land and water managers by providing ready access to sophisticated water-quality models that relate land cover, land use, and point sources of contaminants from specified areas across the land with stream water-quality conditions. By making this capability available over the Internet in a user interface with familiar controls, modelers and water-resource managers alike can experiment with hypothetical scenarios and develop science-based estimates regarding the effects that specific contaminant sources or watershed feature changes may have on water quality. These estimates can then be easily communicated to stakeholders and the general public via the same website. Equally important, the DSS caveats these estimates with model uncertainty to help guide managers to how likely modeled outcomes might occur should modeled management actions be taken.
